# Identifying Mutually Exclusive Gene Sets with Prognostic Value and Novel Potential Driver Genes in Patients with Glioblastoma

**DOI:** 10.1155/2019/4860367

**Published:** 2019-11-05

**Authors:** Qian Gao, Yan Cui, Yanan Shen, Yanyan Li, Xue Gao, Yanfeng Xi, Tong Wang

**Affiliations:** ^1^Department of Health Statistics, Shanxi Medical University, Taiyuan 030001, China; ^2^Tongzhou Center for Disease Prevention and Control, Beijing 101100, China; ^3^Department of Pathology, Shanxi Tumor Hospital, Taiyuan 030013, China

## Abstract

The pathogenesis and prognosis of glioblastoma (GBM) remain poorly understood. Mutual exclusivity analysis can distinguish driver genes and pathways from passenger ones. The purpose of this study was to identify mutually exclusive gene sets (MEGSs) that have prognostic value and to detect novel driver genes in GBM. The genomic alteration profile and clinical information were derived from The Cancer Genome Atlas, and the MEGSA method was used to identify the MEGS. Next, we performed survival analysis and constructed a risk prediction model for prognostic stratification. Leave-one-out cross-validation and permutation test were used to evaluate its performance. Finally, we identified 21 statistically significant MEGSs. We found that the MEGS in the RB pathway was significantly associated with poor prognosis, after adjusting for age and gender (HR = 1.837, 95% CI: 1.192–2.831). Based on the risk prediction model, 208 (80.9%) and 49 (19.1%) patients were assigned to high- and low-risk groups, respectively (log-rank: *p* < 0.001, adjusted *p*=0.001). Additionally, we found that SPTA1, a novel gene involved in the MEGS, was mutually exclusive with members of cell cycle, P53, and RB pathways. In conclusion, the MEGS in the RB pathway had considerable clinical value for GBM prognostic stratification. Mutated SPTA1 may be involved in GBM development.

## 1. Introduction

Glioblastoma (GBM) is the most common and biologically aggressive primary brain tumor [1, 2]. Each year, it affects over 10,000 new patients in the United States [3]. Despite improvements in diagnostic and therapeutic approaches, patients with GBM have poor prognosis. The median overall survival (OS) time is 12–17 months [2, 4–6]. To improve the prognosis of GBM, it is important to understand the carcinogenic mechanism of GBM. Tumor development is primarily driven by the accumulation of lifetime somatic alterations [7, 8]. Therefore, identifying and understanding the genetic and pathway abnormalities that drive the initiation and progression of GBM are critical for the development of effective therapies [2].

The development of the next-generation sequencing has accumulated a large amount of genomic data. The major tasks of analyzing these data are identifying driver alterations that contribute to cancerogenesis and investigating their functional interactions. These tasks can be approached *via* mutual exclusivity (ME) analysis [9, 10]. Mutual exclusivity of genomic alterations, indicating that genes belonging to the same functional pathway tend not to mutate simultaneously in the same patient, has been observed in various cancer types [11, 12]. Over 25% of well-known cancer genes show an mutual exclusivity (ME) pattern [7]. Detecting an ME pattern is important to understand the tumorigenic mechanisms and identify drug targets. Currently, several methods based on mutual exclusivity have been proposed to uncover novel infrequent cancer drivers and investigate their functional relationship [9, 10, 13]. Mutually exclusive gene set analysis (MEGSA), proposed by Hua et al., is a new model to discover mutually exclusive gene sets (MEGSs) from *de novo* or existing biological pathways. Simulation studies have indicated that MEGSA outperformed other methods, such as Dentrix, MDPFinder, Multi-Dentrix, and Mutex, in statistical power and their capability for identifying specific MEGSs, especially for highly imbalanced MEGSs [9]. However, one limitation of this analysis is that only nonsynonymous point mutations were taken into consideration when Hua et al. identified MEGSs in patients with GBM. Consequently, only one mutually exclusive gene pair (PTEN and IDH) was found. Compared with other types of somatic genetic alterations, copy number variation (CNV) accounts for a large fraction of genomic alterations in cancer [14] and plays a critical role in carcinogenesis [14]. Therefore, it is necessary to take CNVs into account when performing mutual exclusivity analysis. Other studies have identified ME patterns related to GBM; however, no study has analyzed their prognostic values [9, 10, 13, 15–20]. Therefore, one purpose of this study is to identify MEGSs and detect novel infrequently driver genes in GBM by integrating nonsynonymous single-nucleotide variants (SNVs) and copy number variations (CNVs) using MEGSA. A further objective is to assess the prognostic value of specific MEGSs.

## 2. Materials and Methods

### 2.1. Data

The preprocessed GBM genomic variant dataset was derived from Multi-Dentrix, which contained 398 alterations (nonsynonymous SNVs and CNVs) and 261 patients [18, 21]. Data preparation for GBM was described in reference [18]. The clinical data were downloaded from The Cancer Genome Atlas (TCGA). Samples with incomplete survival information were excluded, and 257 patients with GBM were enrolled in survival and leave-one-out cross-validation (LOOCV) analysis.

### 2.2. Identifying MEGSs

In this study, the MEGSA was employed to identify MEGSs and novel driver genes. In brief, MEGSA consists of three parts. First, a likelihood ratio (LRT) statistic for testing mutual exclusivity was constructed. Second, global null hypothesis (GNH) analysis was performed to test whether the set of M genes contains an MEGS of any size. Third, the optimal MEGS was identified using model selection [9].

Suppose that *A*_0_ is an MEGS with *N* rows that correspond to patients and *m* columns that represent genes. The entity *a*_*ik*_ denotes the mutation status which is 1 if the gene *k* is mutated for the subject *i* or 0 otherwise. We defined the set of model parameters, Θ=(*γ*, *P*, Π), using coverage, *γ*, gene-specific background mutation rate, Π=(*π*_1_,…, *π*_*m*_), and gene-relative mutation frequencies in *A*_0_, *P*=(*p*_1_,…, *p*_*m*_). Therefore, under the assumption of *p*_*k*_ ∝ *π*_*k*_, the total log likelihood across *N* subjects is defined as

(1) $$\begin{array}{c} \log\;L\left(\gamma ,\Pi ;A_{0}\right)=\overset{N}{\underset{i=1}{\sum }}\log\left(\left(1-\gamma \right)\overset{m}{\underset{k=1}{\prod }}\pi_{k}^{a_{ik}}{\left(1-\pi_{k}\right)}^{1-a_{ik}}+\gamma \frac{1}{\sum_{j=1}^{m}\pi_{j}}\overset{m}{\underset{k=1}{\sum }}\pi_{k}I_{\left\{a_{ik}=1\right\}}\underset{j\neq k}{\prod }\pi_{j}^{a_{ij}}{\left(1-\pi_{j}\right)}^{1-a_{ij}}\right). \end{array}$$

The LRT is calculated as $S=2\left(\log\;L\left({\hat{\gamma }}_{1},{\hat{\Pi }}_{1};A_{0}\right)-\log\;L\left({\hat{\gamma }}_{0},{\hat{\Pi }}_{0};A_{0}\right)\right)$ (*H*_0_ : *γ*=0 versus *H*_1_ : *γ* > 0), with an asymptotically null distribution of 0.5*χ*_0_^2^+0.5*χ*_1_^2^.

The GNH test is completed in three steps: (1) The multiple-path search algorithm is performed to determine the minimum *p* values for gene sets with different size (denoted as *p*_*k*_ (*k*=2,…, *K*)). (2) The permutation test is used to adjust the *p*_*k*_ values and obtain *Q*_*k*_ (*k*=2,…, *K*). Intuitively, *Q*_*k*_ measures the significance that searches only for MEGSs of size *k*. (3) Finally, the overall statistic is defined as *θ*=min(*Q*_2_,…, *Q*_*K*_).

Considering two significant putative MEGSs (*Q*_*k*_ < *θ*_1−*α*_), MEGS1 has two genes (*G*_1_, *G*_2_) with a nominal *p* value of *p*_1_ and MEGS2 has three genes (*G*_1_, *G*_2_, *G*_3_) with a nominal *p* value of *p*_2_ based on LRT. The null hypothesis of model selection would be that none of the M-2 genes (*G*_3_,…, *G*_*M*_) are mutually exclusive of (*G*_1_, *G*_2_). We chose MEGS2 if *p*_2_ < *p*_0_ and *p*_0_ was chosen according to permutations with a false-positive rate <5%. This procedure was repeated until the size of the MEGS reached its preset maximum value, *k*, or the hypothesis test no longer rejected *H*_0_.

### 2.3. Selection and Validation of MEGSs Related to Prognosis

We transformed the gene mutation profile to the MEGS mutation profile by assuming that the MEGS was mutated in a patient if any gene in the gene set was mutated [9]. Univariate and multivariable Cox proportional hazards models were constructed to assess the association between the MEGS, clinical characteristics, and 5-year survival. Next, we developed a risk prediction model based on the prognostic index of the multivariable Cox model for prognostic stratification and evaluated its performance using LOOCV [22, 23]. For each leave-one-out step, the risk score was calculated for the patient who was removed for testing. Following this, each patient was classified into the high- or low-risk group based on whether the risk score was above or below the cut-off value [23]. The cutoff among *N* risk scores was defined using maximally selected log-rank statistics [4, 23, 24]. Survival curves of the high- and low-risk groups were estimated using the Kaplan–Meier method, and significance was assessed using the log-rank test. To overcome any overfitting bias, the permutation test was used to adjust the log-rank *p* value. In brief, we randomly permuted the correspondence of survival time and censoring indicators to covariates and repeated the entire LOOCV process. The adjusted *p* value was calculated as the proportion of permutations whose log-rank statistics were greater than or equal to the value of the statistic for the original data [22, 23].

All analyses were considered statistically significant if *p* < 0.05. All analyses were performed using R 3.3.2 and SAS 9.2.

## 3. Results

### 3.1. MEGSs in GBM

**Figure 1:**
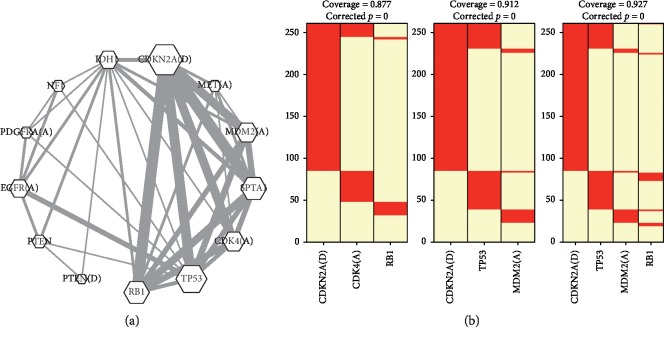
Results of mutual exclusivity analysis. (a) A network constructed based on the 21 significant MEGSs; MET(A) is the abbreviation of the metagene (MET, CAPZA2, ST7, ST7-OT4, ST7-AS1 (A)). (b) The top three most significant MEGSs (*p* < 10^−17^).

*De novo* analyses identified 21 significant but overlapping MEGSs (Supplementary [Supplementary-material supplementary-material-1]). These MEGSs involved 12 genetic abnormalities and a metagene, in which RB1, TP53, IDH1, PTEN, SPTA1, and NF1 occurred as single-nucleotide variants; CDK4, MDM2, EGFR, PDGFRA, and the metagene (MET, CAPZA2, ST7, ST7-AS1, ST7-OT4) possessed copy number amplification; CDKN2A and PTEN possessed copy number deletion.  Figure 1(a) summarizes the 21 significant MEGSs *via* a network construction [9]. The vertexes of the network are genes involved in MEGSs. The edges between gene pairs indicate that these genes are mutually exclusive in at least one MEGS. Furthermore, the weights of vertexes and edges in the network were proportional to the frequency in the detected MEGSs. As shown in Figure 1(a), the most recurrent gene was CDKN2A (14/21), followed by TP53, RB1, CDK4, MDM2, IDH1, EGFR, PTEN, PDGFRA, NF1, and MET. All these genes have been linked to GBM [1, 2, 21, 25, 26]. The top three most significant MEGSs (Figure 1(b)) with *p* < 10^−17^ were core members of the RB (CDK4 amplification, CDKN2A deletion, and RB1 mutations), P53 (CDKN2A deletion, TP53 mutations, and MDM2 amplification), and cell cycle signaling (CDKN2A deletion, RB1 and TP53 mutations, and MDM2 amplification) pathways, respectively [1, 6, 21]. The MEGSs with EGFR amplification and NF1 and TP53 mutations (*p*=1.78 × 10^−7^) were enriched in the MAPK pathway. Compared with other studies, we identified several novel less frequent genes, including SPTA1 (9.6%) and the metagene (MET, CAPZA2, ST7, ST7-OT4, ST7-AS1) (4.6%) [15, 18, 27, 28].

### 3.2. Selection of MEGSs and Clinical Characteristics with Prognostic Value

After excluding individuals with incomplete survival information, 257 patients were enrolled in the prognosis analysis, including 166 (64.6%) males and 91 (35.4%) females. The age at diagnosis ranged from 21 to 89 years with a median of 61 years. The demographics included 234 (91.1%) white patients, and 20 (7.8%) were of other ethnicities (Asian, black, or African American). Of 257 patients, 209 (81.3%) died within 5 years with a median survival time of 14.7 months.

Univariate Cox regression showed age (age ≥50) [4], male, and mutant CDK4(A)/CDKN2A(D)/RB1 and CDK4(A)/SPTA1/RB1/CDKN2A(D) had significant associations with poor prognosis (Table 1 and Supplementary [Supplementary-material supplementary-material-1]). Based on these results, we performed multivariable Cox regression analysis with the stepwise procedure (entry = 0.05, retention = 0.10). These results indicated that age (age ≥50 vs. age <50), gender (male vs. female), and CDK4(A)/CDKN2A(D)/RB1 (mutant vs. wild) were independent prognostic factors (Table 2). After adjusting for age and gender, GBM patients with mutant CDK4(A)/CDKN2A(D)/RB1 had significantly higher risk for 5-year mortality compared with patients with wild type (HR = 1.837, 95% CI: 1.192–2.831).

### 3.3. Prognosis Stratification Based on Risk Prediction Model

**Figure 2:**
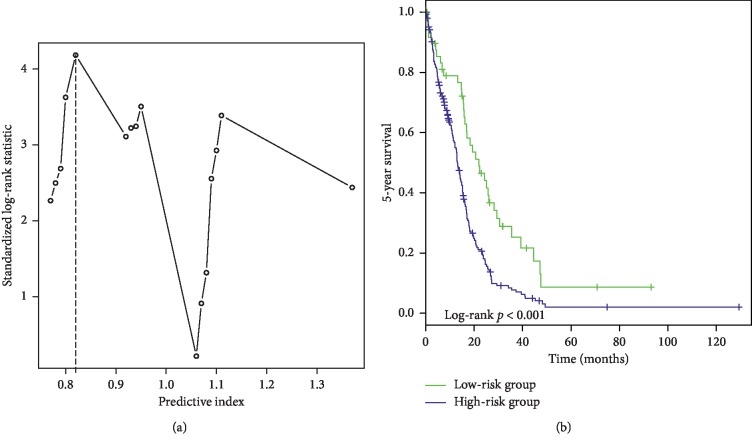
Prognosis stratification based on risk prediction. (a) Identifying the cut-off value using maximally selected log-rank statistics. (b) Kaplan–Meier curves for high- and low-risk groups.

We developed a risk prediction model based on the prognostic index of the multivariable Cox model to divide the patients into low- and high-risk groups. Taking practical and statistical significance into consideration, we chose 0.82 as the cut-off value using the maximally selected log-rank statistics (Figure 2(a)). There were 49 (19.1%) and 208 (80.9%) patients in the low- and high-risk groups, respectively.  Figure 2(b) shows the Kaplan–Meier curves for the low- and high-risk groups (log-rank: *p* < 0.001). The adjusted log-rank *p* value calculated *via* the permutation test (1000 times) was 0.001. The univariate Cox model indicated that the mortality risk within 5 years in the high-risk group was 1.953 times higher than that in the low-risk group (Table 3).

## 4. Discussion

In this study, we identified MEGSs in GBM by integrating nonsynonymous SNVs and CNVs. Most genomic alterations that were involved in MEGSs were enriched in core pathways (RB, P53, and RTK/RAS/PI(3)K pathways) required for GBM pathogenesis [1, 6, 21], providing an important validation for the MEGSA.

The most significant MEGSs included 3 genomic alterations: CDKN2A deletion, CDK4 amplification, and RB1 mutations (covered 87.7%). These genes are core members of the RB pathway, which plays a central role in the regulation of cell proliferation. In quiescent cells, hypophosphorylated RB (active) binds E2F to prevent cell progression through the G1/S cell checkpoint, whereas in the proliferating cell, the D-cyclin/CDK4/6 complex phosphorylates RB (inactive) leading to the release of E2F, which, in turn, induces genes required for DNA synthesis and cell growth. CDKN2A-p16^INK4A^ is a negative regulator of the RB pathway, and CDKN2A-p16^INK4A^ competes with D-cyclins to bind CDK4/6, which prevents the formation of the D-cyclin/CDK4/6 complex [1, 6, 29]. Intuitively, any genomic alteration, including CDKN2A deletion, CDK4 amplification, and RB1 mutations, can inactivate RB, resulting in cell proliferation. Moreover, our results showed that the ME pattern in the RB pathway was associated with poor prognosis in GBM. Previous studies have shown that disrupting the RB pathway is associated with prognosis of various human cancers [30–37]. Immunohistochemical analysis has shown that the underexpressed RB protein in gastric adenocarcinoma [36] and low expression of p16 (encoded by CDKN2A) in oral carcinoma [31], vertical growth phase melanoma [32], esophageal squamous cell carcinoma [35], and GBM [38] significantly predict poor patient survival. Bäcklund et al. have reported that any loss of CDKN2A and RB or the amplification of CDK4 in anaplastic astrocytoma (AA) was associated with decreased survival [39]. Furthermore, poor prognosis in patients with an abnormal RB pathway may be due to high resistance caused by RB silencing to etoposide (VP-16) [40]. An interpretation about mutual exclusivity, referred to as synthetic lethality, is that the secondary driver alteration within the same pathway is detrimental to cells and may result in cell death [12, 13, 41]. Therefore, our study results provided a clue to the development of tumor molecular targeted therapies. Additionally, we developed a risk prediction model for prognosis stratification. The leave-one-out cross-validation and permutation test results revealed the effectiveness of the developed model in our study.

ME analysis can overcome the limitations linked to the frequency-based method for large sample size and detect less frequent mutated genes [9, 10, 13]. Given that CDKN2A, TP53, RB1, PTEN, NF1, CDK4, MDM2, EGFR, PDGFRA, IDH1, and MET are well-known genes associated with GBM, the observed mutual exclusivity suggests that SPTA1 and CAPZA2 may be cancer genes. SPTA1, which is one of the most recurrent genes involved in MEGS, encodes *α*-spectrin. *α*-Spectrin and *ß*-spectrin are assembled into spectrin, which is an actin crosslinking and molecular scaffold protein that determines cell shape and membrane protein location [42, 43]. Alterations in SPTA1 are associated with colorectal cancer [44, 45] and small-cell lung cancer [42]. However, to date, the carcinogenic mechanism of mutated SPTA1 remains unknown. Previous studies have shown that nonerythroid *α*-spectrin interacts with proteins that are related to several cellular processes, such as DNA synthesis, cell cycle progression, and signal transduction, which are consistent with our findings [46]. We found that SPTA1 mutations were mutually exclusive to the core members of the RB, P53, and cell cycle pathways. Taken together, these data indicate that mutated SPTA1 may be related to abnormal cell proliferation and apoptosis in GBM development.

CAPZA2 encodes the human actin-capping protein *α*-subunit. The function of the actin-capping protein is to block the growth of actin filaments by capping the barbed end [47]. The CAZ2 protein is overexpressed in breast cancer, and the F-actin-capping protein is linked to renal cell carcinoma [48, 49]. Moreover, Mueller et al. have observed CAPZA2 amplification in glioma, which was in good agreement with our observation [50]. However, the investigations about the role of CAPZA2 in cancer are rare. It is possible that CAPZA2 may play a role in tumor-specified cell motility [48]. Recently, Ohishi et al. found that CAPZA2 negatively regulates cell invasion [51], which indicates that amplified CAPZA2 may be a favorable prognosis marker in cancer. To explore the prognostic value of SPTA1 and CAPZA2, survival analyses were performed. However, our results showed that neither SPTA1 (*p*=0.764) nor CAPZA2 (*p*=0.213) had significant associations with survival in patients with GBM after adjusting for age, gender, and CDK4(A)/RB1/CDKN2A(D). These data suggest that alterations in SPTA1 and CAPZA2 may be linked to the formation of GBM alone. Nonetheless, the mutually exclusive gene set CDK4(A)/CDKN2A(D)/RB1 was involved in the formation of GBM and predicted the prognosis of GBM.

The main limitation of this study was the lack of external validation, which makes our results less reliable. Therefore, a study using a larger patient cohort and an experiment with cell lines are required to validate our findings and allow more reliable conclusions to be reached.

## 5. Conclusions

In summary, we derived 21 MEGSs by integrating nonsynonymous single-nucleotide variants and copy number variations. In these MEGSs, only the ME pattern in the RB pathway predicted the prognosis of patients with GBM after adjusting for age and gender. This finding may help researchers develop molecular targeted therapies and identify high-risk GBM for better treatment. Additionally, we obtained several less frequent cancer genes, which may extend our knowledge on the pathogenesis of GBM.

## Figures and Tables

**Table 1:** Significant factors in univariate survival analysis.

Factors		*N* (%)	$\hat{\beta }$	SE ($\hat{\beta }$)	Wald *χ*^2^	*p*	HR (95% CI)
Age	≥50	215 (83.66)	0.540	0.195	7.62	**0.006**	1.716 (1.117, 2.516)
	<50	42 (16.34)					

Gender	Male	166 (64.59)	0.312	0.148	4.44	**0.035**	1.366 (1.022, 1.826)
	Female	91 (35.41)					

CDK4(A)/RB1/CDKN2A(D)	Mutant	225 (87.55)	0.587	0.219	7.20	**0.007**	1.799 (1.171, 2.761)
	Wild	32 (14.22)					

CDK4(A)/SPTA1/RB1/CDKN2A(D)	Mutant	228 (88.72)	0.571	0.227	6.30	**0.012**	1.769 (1.133, 2.762)
	Wild	29 (11.28)					

**Table 2:** Results of multivariable Cox proportional hazards analysis.

Variables	$\hat{\beta }$	SE ($\hat{\beta }$)	Wald *χ*^2^	*p*	HR (95% CI)
Age	0.455	0.197	5.31	0.021	1.576 (1.070, 2.319)
Gender	0.325	0.151	4.67	0.031	1.384 (1.031, 1.859)
CDK4(A)/CDKN2A(D)/RB1	0.608	0.221	7.59	0.006	1.837 (1.192, 2.831)

**Table 3:** Cox regression containing only group variable.

Variables	$\hat{\beta }$	SE ($\hat{\beta }$)	Wald *χ*^2^	*p*	HR (95% CI)
Class	0.669	0.185	13.04	0.000305	1.953 (1.358, 2.809)

## Data Availability

The results published here are based on the data generated by the TCGA database at http://cancergenome.nih.gov/. The preprocessed GBM genomic variant dataset was derived from the study by Leiserson et al. [18].

## Supplementary Materials

Supplementary Materials Supplementary Table S1: MEGSs identified by MEGSA. Supplementary Table S2: univariate Cox proportional hazards analysis.
