# Supplementary material for: Identifying Mutually Exclusive Gene Sets with Prognostic Value and Novel Potential Driver Genes in Patients with Glioblastoma
Source: Biomed Res Int. 2019 Nov 5;2019:4860367. doi: 10.1155/2019/4860367 (PMC6878817; doi:10.1155/2019/4860367)
Supplement: Supplementary Materials — Supplementary Table S1: MEGSs identified by MEGSA. Supplementary Table S2: univariate Cox proportional hazards analysis. [file 4860367.f1.zip › 4860367.f1/Table S1.docx]

**T****able S1** MEGS identified by MEGSA

| **MEGS** | **Coverage** | **LTR** | **$P$** | **q^a^** |
| --- | --- | --- | --- | --- |
| CDK4(A),CDKN2A(D),RB1**^b^** | 87.7% | 80.48 | **1.47e-19** | <0.001 |
| CDKN2A(D),TP53,MDM2(A) | 91.2% | 75.48 | **1.84e-18** | <0.001 |
| CDKN2A(D),RB1,MDM2(A), TP53 | 92.7% | 72.27 | **9.39e-18** | <0.001 |
| CDKN2A(D),RB1,TP53,CDK4(A) | 94.6% | 66.09 | 2.15e-16 | <0.001 |
| CDKN2A(D), SPTA1, MDM2(A),TP53 | 92.7% | 65.84 | 2.45e-16 | <0.001 |
| CDKN2A(D),SPTA1,MDM2(A),IDH1,TP53 | 93.9% | 65.58 | 2.79e-16 | <0.001 |
| CDKN2A(D),SPTA1,MDM2(A),IDH1,RB1 | 87.4% | 63.33 | 8.72e-16 | <0.001 |
| CDKN2A(D), SPTA1, MDM2(A), RB1, TP53 | 93.9% | 61.87 | 1.83e-15 | <0.001 |
| CDKN2A(D),SPTA1,RB1,CDK4(A)  [MET,CAPZA2,ST7-AS1,ST7,ST7-OT4(A)] | 90.4% | 60.12 | 4.47e-15 | <0.001 |
| CDKN2A(D), SPTA1,RB1,IDH1,CDK4(A) | 90.4% | 58.16 | 1.21e-14 | <0.001 |
| CDKN2A(D),SPTA1,CDK4(A),RB1 | 88.9% | 56.10 | 3.45e-14 | <0.001 |
| CDK4(A),CDKN2A(D),TP53 | 92.3% | 54.80 | 6.66e-14 | <0.001 |
| CDKN2A(D),SPTA1,RB1,MDM2(A)  [MET,CAPZA2,ST7-AS1,ST7,ST7-OT4(A)] | 86.2% | 53.29 | 1.44e-13 | <0.001 |
| CDKN2A(D), SPTA1, CDK4(A),TP53 | 93.4% | 46.07 | 5.69e-12 | <0.001 |
| EGFR(A),PDGFRA(A),NF1,IDH1 | 72.0% | 35.86 | 1.06e-09 | 0.009 |
| PTEN, PTEN(D), IDH1 | 49.4% | 33.08 | 4.42e-09 | <0.001 |
| EGFR(A) TP53 | 71.3% | 31.04 | 1.26e-8 | <0.001 |
| EGFR(A), PDGFRA(A), TP53 | 78.9% | 29.87 | 2.32e-08 | <0.001 |
| EGFR(A),NF1,TP53 | 74.3% | 25.92 | 1.78e-07 | 0.013 |
| EGFR(A),PTEN,IDH1 | 72.8% | 24.36 | 3.99e-07 | 0.046 |
| EGFR(A),PTEN,TP53 | 82.4% | 24.19 | 4.36e-07 | 0.046 |

**^a^**q: adjusted p value

**^b^**The characters in parentheses behind the name of gene indicate the copy number variations: (D) means deletion, (A) means amplification.
