# Supplementary material for: Identifying Mutually Exclusive Gene Sets with Prognostic Value and Novel Potential Driver Genes in Patients with Glioblastoma
Source: Biomed Res Int. 2019 Nov 5;2019:4860367. doi: 10.1155/2019/4860367 (PMC6878817; doi:10.1155/2019/4860367)
Supplement: Supplementary Materials — Supplementary Table S1: MEGSs identified by MEGSA. Supplementary Table S2: univariate Cox proportional hazards analysis. [file 4860367.f1.zip › 4860367.f1/Table S2.docx]

**Table S2** Univariate Cox proportional hazards analysis

| Factors |  | N (%) | $\hat{\beta}$ | SE($\hat{\beta}$) | $Wald\text{ }\chi^{2}$ | P | HR  (95% CI) |
| --- | --- | --- | --- | --- | --- | --- | --- |
| age^a^ | ≥50 | 215(83.66) | 0.540 | 0.195 | 7.62 | **0.006** | 1.716  (1.117,2.516) |
|  | <50 | 42(16.34) |  |  |  |  |  |
| Gender | Male | 166(64.59) | 0.312 | 0.148 | 4.44 | **0.035** | 1.366  (1.022,1.826) |
|  | Female | 91(35.41) |  |  |  |  |  |
| race | white  others  missing | 234(91.05)  20(7.78)  3(1.17) | 0.299 | 0.310 | 0.93 | 0.335 | 1.348  (0.734,2.476) |
| CDK4(A)/RB1/  CDKN2A(D) | Mutant  Wild | 225(87.55)  32(14.22) | 0.587 | 0.219 | 7.20 | **0.007** | 1.799  (1.171,2.761) |
| CDKN2A(D)/TP53/  MDM2(A) | Mutant  Wild | 234(91.05)  23(8.95) | 0.157 | 0.241 | 0.42 | 0.515 | 1.170  (0.729,1.877) |
| CDKN2A(D)/RB1/  MDM2(A)/TP53 | Mutant  Wild | 238(92.61)  19(7.39) | 0.235 | 0.261 | 0.81 | 0.367 | 1.265  (0.759,2.110) |
| CDKN2A(D)/RB1/  TP53/CDK4(A) | Mutant  Wild | 243(94.55)  14(5.45) | 0.265 | 0.298 | 0.79 | 0.373 | 1.304  (0.727,2.339) |
| CDKN2A(D)/SPTA1/ MDM2(A)/TP53 | Mutant  Wild | 238(92.61)  19(7.39) | 0.123 | 0.261 | 0.22 | 0.638 | 1.131  (0.678,1.884) |
| CDKN2A(D)/SPTA1/  MDM2(A)/IDH1/TP53 | Mutant  Wild | 241(93.77)  16(6.23) | -0.075 | 0.278 | 0.07 | 0.787 | 0.928  (0.539,1.598) |
| CDKN2A(D)/SPTA1/  MDM2(A)/IDH1/RB1 | Mutant  Wild | 224(87.16)  33(12.84) | 0.211 | 0.210 | 1.01 | 0.315 | 1.235  (0.818,1.865) |
| CDKN2A(D)/SPTA1/ MDM2(A)/RB1/TP53 | Mutant  Wild | 241(93.77)  16(6.23) | 0.159 | 0.277 | 0.33 | 0.568 | 1.172  (0.680,2.018) |
| CDKN2A(D)/SPTA1/  RB1/CDK4(A)/MET(A)^b^ | Mutant  Wild | 232(90.27)  25(10.78) | 0.241 | 0.231 | 1.09 | 0.296 | 1.273  (0.810,2.000) |
| CDKN2A(D)/SPTA1/  RB1/IDH1/CDK4(A) | Mutant  Wild | 232(90.27)  25(10.78) | 0.310 | 0.231 | 1.80 | 0.179 | 1.364  (0.867,2.144) |
| CDK4(A)/SPTA1/RB1/ CDKN2A(D) | Mutant  Wild | 228(88.72)  29(11.28) | 0.571 | 0.227 | 6.30 | **0.012** | 1.769  (1.133,2.762) |
| CDK4(A)/CDKN2A(D)/  TP53 | Mutant  Wild | 237(92.22)  20(7.78) | 0.118 | 0.261 | 0.20 | 0.651 | 1.125  (0.675,1.876) |
| CDKN2A(D)/SPTA1/  RB1/MDM2(A)/  MET(A) | Mutant  Wild | 221(85.99)  36(14.01) | 0.242 | 0.201 | 1.45 | 0.228 | 1.274  (0.859,1.888) |
| CDKN2A(D)/SPTA1/ CDK4(A)/TP53 | Mutant  Wild | 240(93.39)  17(6.61) | -0.0008 | 0.277 | 0 | 1 | 0.999  (0.580,1.721) |
| EGFR(A)/PDGFRA(A)/  NF1/IDH1 | Mutant  Wild | 185(71.98)  72(28.02) | -0.057 | 0.153 | 0.14 | 0.708 | 0.944  (0.699,1.275) |
| PTEN/PTEN(D)/IDH1 | Mutant  Wild | 126(49.03)  131(50.97) | -0.174 | 0.139 | 1.56 | 0.211 | 0.840  (0.640,1.104) |
| EGFR(A)/TP53 | Mutant  Wild | 183(71.21)  74(28.79) | -0.148 | 0.150 | 0.97 | 0.326 | 0.863  (0.643,1.158) |
| EGFR(A)/PDGFRA(A)/ TP53 | Mutant  Wild | 203(78.99)  54(21.01) | -0.166 | 0.165 | 1.01 | 0.315 | 0.847  (0.613,1.171) |
| EGFR(A)/NF1/TP53 | Mutant  Wild | 191(74.32)  66(25.68) | -0.168 | 0.156 | 1.16 | 0.282 | 0.845  (0.623,1.148) |
| EGFR(A)/PTEN/IDH1 | Mutant  Wild | 186(72.37)  71(27.63) | -0.072 | 0.153 | 0.22 | 0.641 | 0.931  (0.690,1.257) |
| EGFR(A)/PTEN/TP53 | Mutant  Wild | 211(82.10)  46(17.90) | -0.190 | 0.173 | 1.20 | 0.273 | 0.827  (0.589,1.161) |

**^a^** Boldface indicates that p-value is less than significant level 0.05.

**^b^**MET(A) is the abbreviation of meta gene[*MET, CAPZA2, ST7, ST7-OT4, ST7-AS1* (A)]
